# Non–English Language Preference and Breast Cancer Outcomes

**DOI:** 10.1001/jamanetworkopen.2025.14036

**Published:** 2025-06-05

**Authors:** Daphna Y. Spiegel, Josephine Levey, Anna Modest, Julia Willcox, Nisha Bhargava, Ranjna Sharma, Abram Recht

**Affiliations:** 1 Department of Radiation Oncology, Beth Israel Deaconess Medical Center and Harvard Medical School, Boston, Massachusetts; 2 Department of Radiation Oncology, Beth Israel Deaconess Medical Center, Boston, Massachusetts; 3 Department of Obstetrics and Gynecology, Beth Israel Deaconess Medical Center and Harvard Medical School, Boston, Massachusetts; 4 Department of Surgery, Beth Israel Deaconess Medical Center and Harvard Medical School, Boston, Massachusetts

## Abstract

**Importance:**

Non–English language preference (NELP) poses a challenge in health care delivery and may contribute to disparate outcomes. Understanding the impact of NELP can provide insight for health care professionals, policymakers, and advocates aiming to enhance care quality for diverse populations.

**Objective:**

To investigate the association of NELP with breast cancer–related outcomes.

**Design, Setting, and Participants:**

This single-center cohort study of patients with breast cancer treated with curative intent was performed at a tertiary academic medical center from January 1, 2000, to December 31, 2020. A total of 22 355 patients were included in the initial cohort acquired through electronic medical record data; 2261 had complete data when cross-referenced with the institutional cancer registry. Data were analyzed from January 8 to December 11, 2024.

**Exposure:**

Language preference.

**Main Outcomes and Measures:**

The primary end point was disease-specific survival (DSS) rates for patients with NELP compared with patients with English language preference (ELP), with adjustment for baseline clinical and demographic characteristics and treatments. Secondary end points included disease-free survival (DFS) and overall survival (OS). Time from diagnosis to definitive surgery was also analyzed. Kaplan-Meier analyses and log-rank tests were used to compare survival in the NELP and ELP groups.

**Results:**

The 2261 patients included in the analysis consisted of 2023 with ELP and 238 with NELP (2240 female [99.1%]; mean [SD] age, 59.6 [12.3] years). Clinical and treatment data were well balanced between the NELP and ELP groups. Median follow-up was 60.0 (IQR, 40.0-76.6) months. Median time between biopsy and definitive surgery was significantly longer for the NELP group compared with the ELP group (49 [IQR, 29-75] vs 38 [IQR, 24-57] days; *P* < .001). Stratified by insurance, only those with Medicare had significant delays between biopsy and surgery (median, 54 [IQR, 29-96] vs 36 [IQR, 25-53] days; *P* < .001). Five-year DSS rates for the NELP and ELP groups were 98.5% (95% CI, 93.8%-99.6%) and 99.0% (95% CI, 98.3%-99.4%), respectively (log-rank *P* = .39). DFS was 93.9% (95% CI, 89.4%-96.5%) for the NELP group and 95.6% (95% CI, 94.4%-96.5%) for the ELP group (log-rank *P* = .96). OS was 94.4% (95% CI, 89.3%-97.1%) for the NELP group and 96.7% (95% CI, 95.7%-97.5%) for the ELP group (log-rank *P* = .26).

**Conclusions and Relevance:**

In this cohort study examining the association of NELP with breast cancer outcomes, patients with NELP had significant delays in definitive surgical management compared with patients with ELP, but DSS, DFS, and OS did not differ significantly between groups. This suggests the importance of having robust support systems to mitigate language-related disparities and preserve breast cancer outcomes in this vulnerable population.

## Key Points

**Question:**

Is non–English language preference (NELP) associated with breast cancer outcomes?

**Findings:**

This cohort study of 2261 patients with breast cancer treated at an academic medical center found that NELP was not associated with differences in the primary outcome of disease-specific survival or other survival outcomes. However, NELP was associated with a significant delay in surgical management.

**Meaning:**

These findings suggest that, while NELP may not be directly associated with survival outcomes in a resource-rich setting, targeted interventions are needed to address treatment delays and socioeconomic disparities for patients with NELP.

## Teaser

This cohort study investigates whether non–English language preference is independently associated with survival among patients with breast cancer.

## Introduction

Many factors influence a patient’s ability to access preventive breast cancer screening and receive timely treatment, including race, ethnicity, health literacy, immigration status, and other social determinants of health.^1,2,3,4^ Such barriers to care for minoritized and underserved populations are increasingly recognized, but the impact of non–English language preference (NELP) on cancer outcomes remains underexplored.^2,5,6,7^ English proficiency is essential not only for direct communication with health care professionals but also for navigating the complexities of the US health care system. These communication challenges can exacerbate existing disparities in care and potentially lead to worse outcomes.^8,9,10,11,12^ In fact, prior work has shown that NELP is associated with inferior local control in patients with head and neck cancers compared with patients with English language preference (ELP), underscoring the critical need to address this gap.^13^

Language barriers are especially pertinent for patients with breast cancer, whose treatment often involves prolonged, multifaceted interactions with the health care system. Individuals with NELP are less likely to undergo breast cancer screening.^3,14^ Further, a survey of more than 700 breast care centers across the US found that 69% reported mammography results exclusively in English.^15^ Additionally, even after receiving appropriate counseling, patients with NELP are more likely to opt for alternative treatments rather than the standard recommendations from a multidisciplinary tumor board discussion.^11^

Understanding the association of NELP with breast cancer outcomes is therefore crucial. This study investigates whether NELP is independently associated with breast cancer outcomes. We hypothesized that patients with NELP would have worse cancer outcomes compared with those with ELP, even when adjusted for baseline clinical and demographic characteristics and treatments. Identifying these potential differences in care related to NELP could ultimately highlight areas for improvement in the health care system, inform targeted interventions aimed at reducing treatment disparities, and improve cancer-related outcomes.

## Methods

### Study Population

We performed a retrospective analysis of patients with breast cancer treated with curative intent at a single academic medical center from January 1, 2000, to December 31, 2020. A total of 22 355 patients were included in the initial patient cohort acquired through extraction of electronic medical record (EMR) data. This group was cross-referenced with the institutional cancer registry, resulting in a final study population of 2261 patients with complete demographic, clinical, and follow-up data. Patients self-reported demographic information, including race, ethnicity, and language preference. Race was categorized as African American or Black, American Indian or Alaska Native, Asian, White, or other and ethnicity as Hispanic or non-Hispanic. Race and ethnicity were included as covariates, as these can directly influence language preferences. Language preference was documented during an initial hospital registration call, when patients were asked about their preferred language and interpreter needs. Patients were classified as having NELP if they self-reported a preference for conducting visits in a language other than English and requested an interpreter for medical visits. If patients expressed a preference for an interpreter, an interpreter joined the registration call. Both spoken and written language preferences were also routinely recorded in the EMR. Clinicians maintain the ability to update both language and interpreter preferences in the EMR system, should patients’ needs or preferences shift over time. This study was approved by the Institutional Review Board of Beth Israel Deaconess Medical Center, with a waiver or exemption of consent because the study was of minimal risk to patients and did not involve direct patient contact. This study followed the Strengthening the Reporting of Observational Studies in Epidemiology (STROBE) reporting guidelines for cohort studies.

### End Points

The primary end point was the disease-specific survival (DSS) rate. Secondary end points included disease-free survival (DFS) and overall survival (OS) rates. DSS was defined as the time from the date of diagnosis to the date of death from breast cancer or the last follow-up appointment. DFS was defined as the date of diagnosis to the date of recurrence or death. OS was defined as the date of diagnosis to the date of last follow-up or the date of death. Recurrence was defined as disease occurring following definitive treatment confirmed by tissue biopsy, and time to recurrence was defined as the date of diagnosis to the date of subsequent biopsy that confirmed disease recurrence

### Statistical Analysis

Data were analyzed from January 8 to December 11, 2024. Data are presented as mean (SD), median (IQR), or number (percentage). Median follow-up time in months was calculated using the reverse Kaplan-Meier method and is presented as median (IQR).^16^ Categorical variables were compared using χ^2^ or Fisher exact tests. Continuous variables were compared using the Wilcoxon rank sum test. Median follow-up and survival times were compared using the log-rank test. Hazard ratios (HRs) and 95% CIs were calculated using the Cox proportional hazards model after ensuring model assumptions. Confounders were determined a priori or if there were differences between the NELP and ELP groups at baseline. All survival models were adjusted for marital status, insurance, smoking, Charlson Comorbidity Index score, race, and ethnicity.

Several subgroup analyses were conducted. Using the patient’s residential zip code at the time of treatment, individuals were classified as living in low-income neighborhoods if the mean income in their zip code was 10% or more below the federal poverty level. Zip code was also used to determine whether the neighborhood had low educational attainment, defined as less than 50% of residents with some college or an associate’s degree. Additionally, to assess the association of the interval from biopsy to definitive surgery with the outcome, we analyzed recurrence and survival among those with a gap from biopsy to definitive surgery of 60 days or more. Sixty days was selected based on the position statement from the American Society of Breast Surgeons, which states that all patients should initiate treatment for breast cancer within 60 days of the first diagnostic biopsy.^17^ Finally, a sensitivity analysis was conducted to account for additional support provided to Mandarin- and Cantonese-speaking patients, as all Chinese-speaking patients with breast cancer had access to a culturally specific patient navigator throughout the study period. All data were analyzed using SAS, version 9.4 (SAS Institute Inc) and 2-sided *P* < .05 was considered statistically significant.

## Results

### Patient, Disease, and Treatment Characteristics

The final analysis included 2261 patients, of whom 2240 (99.1%) were female and 21 (0.9%) were male, with a mean (SD) age of 59.6 (12.3) years. In terms of race and ethnicity, 253 patients (11.2%) identified as African American or Black; 1 (0.04%), American Indian or Alaskan Native; 210 (9.3%), Asian; 1611 (71.3%), White; 186 (8.2%), other race; and 110 (4.9%), Hispanic. The overall study population consisted of 2023 patients in the ELP group and 238 in the NELP group. Among the NELP cohort, 101 patients (42.4%) preferred Mandarin or Cantonese Chinese, 48 (20.2%) preferred Spanish, 25 (10.5%) preferred Russian, 10 (4.2%) preferred Portuguese, and 54 (22.7%) preferred other languages (Figure). Patient characteristics in these 2 cohorts are shown in Table 1. The mean (SD) age at diagnosis was similar for both groups: 59.6 (12.3) years for NELP and 59.7 (12.3) years for ELP. The NELP cohort was more diverse in terms of race and ethnicity, with higher proportions of Asian (119 [50.0%] vs 91 [4.5%]) and Hispanic (49 [20.6%] vs 61 [3.0%]) patients compared with the ELP group. Patients with NELP were more likely to have Medicaid coverage (123 [51.7%] vs 210 [10.4%]) and less likely to have private insurance (36 [15.1%] vs 1049 [51.9%]). Disease and treatment characteristics were well-balanced between the NELP and ELP patient groups (Table 2 and Table 3) and not included in further adjusted models.

**Figure.**
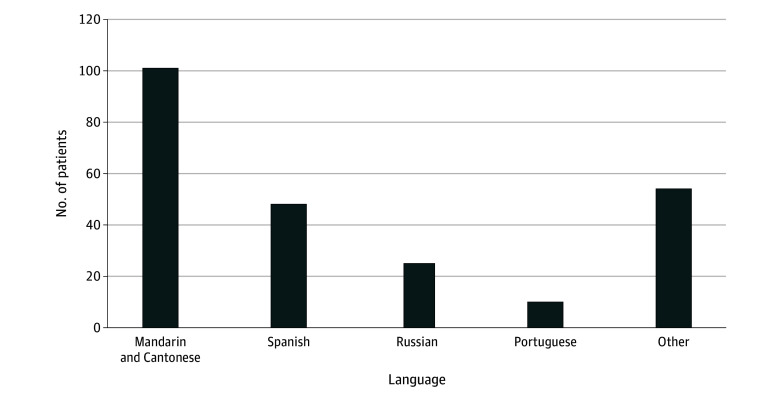
Preferred Language Distribution Among Patients With Non–English Language Preference

**Table 1.** Patient Characteristics

Characteristic	Patient group, No. (%)	
	NELP (n = 238)	ELP (n = 2023)
Age at diagnosis, mean (SD), y	59.6 (12.3)	59.7 (12.3)
Sex		
Female	237 (99.6)	2003 (99.0)
Male	1 (0.4)	20 (1.0)
Race		
African American or Black	22 (9.2)	231 (11.4)
American Indian or Alaska Native	0	1 (0.05)
Asian	119 (50.0)	91 (4.5)
White	58 (24.4)	1553 (76.8)
Other	39 (16.4)	147 (7.3)
Hispanic		
Yes	49 (20.6)	61 (3.0)
No	175 (73.5)	1792 (88.6)
Unknown or refused	14 (5.9)	170 (8.4)
Marital status		
Single	39 (16.4)	444 (21.9)
Married	149 (62.6)	1169 (57.8)
Other	50 (21.0)	410 (20.3)
Insurance		
Medicare	79 (33.2)	757 (37.4)
Medicaid	123 (51.7)	210 (10.4)
Private	36 (15.1)	1049 (51.9)
Self-pay	0	7 (0.3)
Smoking status		
Never	144 (60.5)	794 (39.2)
Current	4 (1.7)	61 (3.0)
Former	9 (3.8)	358 (17.7)
Unknown	81 (34.0)	810 (40.0)
Charlson Comorbidity Index score		
0-2	159 (66.8)	1381 (68.3)
3-5	59 (24.8)	565 (27.9)
6-14	20 (8.4)	77 (3.8)

Abbreviations: ELP, English language preference; NELP, non–English language preference.

**Table 2.** Disease Features

Feature	Patient group, No. (%)	
	NELP (n = 238)	ELP (n = 2023)
AJCC stage		
0	43 (18.1)	267 (13.2)
IA-IB	131 (55.0)	1326 (65.5)
IIA-IIB	55 (23.1)	327 (16.2)
IIIA-IIIC	9 (3.8)	101 (5.0)
Missing	0	2 (0.1)
Grade		
1	51 (21.4)	490 (24.2)
2	110 (46.2)	925 (45.7)
3	75 (31.5)	591 (29.2)
Missing	2 (0.8)	17 (0.8)
Histology		
DCIS	38 (16.0)	225 (11.1)
IDC	141 (59.2)	1177 (58.2)
ILC	16 (6.7)	239 (11.8)
Mixed IDC/ILC	26 (10.9)	285 (14.1)
Other	10 (4.2)	66 (3.3)
Unknown	7 (2.9)	31 (1.5)
ER status		
Positive	206 (86.6)	1761 (87.0)
Negative	31 (13.0)	257 (12.7)
Missing	1 (0.4)	5 (0.2)
PR status		
Positive	135 (56.7)	1290 (63.8)
Negative	57 (23.9)	458 (22.6)
Not tested	46 (19.3)	271 (13.4)
Missing	0	4 (0.2)
*ERBB2* (formerly *HER2*) status		
Positive	25 (10.5)	197 (9.7)
Negative	166 (69.7)	1560 (77.1)
Not tested	46 (19.3)	265 (13.1)
Missing	1 (0.4)	1 (0.05)

Abbreviations: AJCC, American Joint Committee on Cancer; DCIS, ductal carcinoma in situ; ELP, English language preference; ER, estrogen receptor; IDC, invasive ductal carcinoma; ILC, invasive lobular carcinoma; NELP, non–English language preference; PR, progesterone receptor.

**Table 3.** Treatment Characteristics

Characteristic	Patient group, No. (%)	
	NELP (n = 238)	ELP (n = 2023)
Type of breast surgery		
BCS	173 (72.7)	1448 (71.6)
Mastectomy	62 (26.1)	562 (27.8)
Missing	3 (1.3)	13 (0.6)
Type of axillary surgery		
SLNB only	165 (69.3)	1490 (73.7)
ALND	21 (8.8)	118 (5.8)
SLNB followed by ALND	3 (1.3)	67 (3.3)
Targeted axillary excision plus SLNB	0	7 (0.3)
None	47 (19.7)	332 (16.4)
Missing	2 (0.8)	9 (0.4)
Chemotherapy		
Yes	78 (32.8)	649 (32.1)
No	160 (67.2)	1374 (67.9)
Endocrine therapy		
Yes	191 (80.3)	1606 (79.4)
No	46 (19.3)	417 (20.6)
Missing	1 (0.4)	0
Radiation therapy		
Yes	159 (66.8)	1366 (67.5)
No	79 (33.2)	657 (32.5)

Abbreviations: ALND, axillary lymph node dissection; BCS, breast-conserving surgery; ELP, English language preference; NELP, non–English language preference; SLNB, sentinel lymph node biopsy.

### Survival Outcomes

Median follow-up was 60.0 (IQR, 40.0-76.6) months. Five-year DSS rates were 98.5% (95% CI, 93.8%-99.6%) in the NELP cohort and 99.0% (95% CI, 98.3%-99.4%) in the ELP cohort (log-rank *P* = .39), with an adjusted HR of 0.79 (95% CI, 0.21-2.90). Five-year DFS rates were 93.9% (95% CI, 89.4%-96.5%) in the NELP cohort and 95.6% (95% CI, 94.4%-96.5%) in the ELP cohort (log-rank *P* = .96), with an adjusted HR of 0.85 (95% CI, 0.41-1.70). Five-year OS rates were 94.4% (95% CI, 89.3%-97.1%) in the NELP cohort and 96.7% (95% CI, 95.7%-97.5%) in the ELP cohort (log-rank *P* = .26), with an adjusted HR of 0.96 (95% CI, 0.42-2.20). In an additional sensitivity analysis that did not include Mandarin- and Cantonese-speaking patients in the NELP cohort due to the availability of a culturally specific patient navigator, survival outcomes remained comparable between this NELP subgroup and the ELP group. The 5-year DSS rates were 97.3% (95% CI, 89.5%-99.3%) vs 99.0% (95% CI, 98.3%-99.4%; adjusted HR, 0.66; 95% CI, 0.19-2.30), DFS rates were 90.1% (95% CI, 82.6%-94.5%) vs 95.6% (95% CI, 94.4%-96.5%; adjusted HR, 0.72; 95% CI, 0.36-1.40), and OS rates were 93.9% (95% CI, 86.5%-97.3%) vs 96.7% (95% CI, 95.7%-97.5%; adjusted HR, 0.91; 95% CI, 0.37-2.20).

### Time to Surgery

The time from biopsy to definitive surgery was significantly longer for patients with NELP (median, 49 [IQR, 29-75] days) compared with patients with ELP (38 [IQR, 24-57] days; *P* < .001). Patients with Medicare insurance and NELP had longer times from diagnosis to surgery compared with patients with ELP with Medicare insurance (54 [IQR, 29-96] vs 36 [IQR, 25-53] days; *P* < .001). There was no difference in time to surgery for the NELP compared with the ELP groups for those with Medicaid or private insurance. Regardless of neighborhood income level, patients with NELP experienced longer times to surgery compared with patients with ELP, with median time to surgery for patients with NELP in low-income neighborhoods at 50 (IQR, 28-86) days compared with 37 (IQR, 23-59) days for patients with ELP (*P* = .001) and median time to surgery for patients with NELP in high-income neighborhoods at 49 (IQR, 29-67) days compared with 38 (IQR, 24-56) days for patients with ELP (*P* = .01). There was no statistically significant difference in time to surgery for patients with NELP compared with patients with ELP in the subgroups with low educational attainment; however, there was increased median time to surgery for patients with NELP compared with ELP in the subgroups with high educational attainment (49 [IQR, 29-72] vs 38 [IQR, 24-56] days; *P* < .001) (Table 4). In our additional sensitivity analysis separately analyzing patients with NELP who preferred non-English languages other than Chinese, median time to surgery remained significantly longer for the NELP cohort compared with the ELP cohort (49 [IQR, 28-91] vs 38 [IQR, 24-57] days; *P* < .001).

**Table 4.** Time to Surgery

	Patient group, median (IQR), d		
	NELP (n = 238)	ELP (n = 2024)	*P* value
Time from diagnosis to surgery	49 (29-75)	38 (24-57)	<.001
Stratified by insurance			
Medicare	54 (29-96)	36 (25-53)	<.001
Medicaid	47 (29-69)	47 (28-70)	.88
Private	49 (22-70)	37 (23-57)	.24
Stratified by neighborhood income^a^			
Low	50 (28-86)	37 (23-59)	.001
No. of patients	141	630	NA
High	49 (29-67)	38 (24-56)	.01
No. of patients	97	1393	NA
Stratified by educational attainmen^b^			
Low	50 (17-100)	41 (26-60)	.48
No. of patients	23	108	NA
High	49 (29-72)	38 (24-56)	<.001
No. pf patients	215	1915	NA

Abbreviations: ELP, English language preference; NA, not applicable; NELP, non–English language preference.

^a^ Patients were classified as living in low-income neighborhoods if the mean income in their zip code was 10% or more below the federal poverty level and as living in high-income neighborhoods if the mean income in their zip code was more than 10% above the federal poverty level.

^b^ Using the patient's residential zip code at the time of treatment, low educational attainment was defined as less than 50% of residents with some college or an associate's degree and high educational attainment as 50% or more of residents with some college or an associate’s degree.

We also examined the association of the interval from biopsy to definitive surgery with outcomes for each cohort. There were no differences in crude recurrence rates (6 [6.8%] vs 32 [6.9%]; *P* = .98) or breast cancer–related deaths (3 [3.4%] vs 8 [1.7%]; *P* = .39) for patients with NELP compared with ELP with a biopsy-to-surgery interval of 60 days or more. Patients in the NELP and ELP groups with intervals from biopsy to surgery of less than 60 days had similar crude recurrence rates (7 [4.7%] vs 96 [6.2%]; *P* = .46) and cancer-related deaths (1 [0.7%] vs 15 [1.0%]; *P* = .72).

## Discussion

This study evaluated the association of NELP with breast cancer outcomes in a large cohort of patients treated at a tertiary academic medical center. After adjusting for clinical and demographic factors, we found no significant differences in DSS, DFS, or OS between the NELP and ELP groups. These findings suggest that NELP itself may not be independently associated with breast cancer survival when adequate support systems and institutional resources are in place to address language barriers. However, our analysis also revealed disparities in socioeconomic factors and delays in treatment initiation among patients in the NELP group, highlighting the need for targeted interventions to mitigate these challenges.

The absence of a significant survival disparity between the NELP and ELP groups may reflect the institution’s efforts to address the needs of patients with NELP, such as providing comprehensive interpreter services and culturally tailored patient navigation programs. For the duration of the study period, a culturally specific patient navigator was provided for all Chinese language–speaking patients with breast cancer. This employee was part of the Cancer Center Social Work department and spoke Mandarin and Cantonese Chinese, the primary languages of the largest percentage of patients with NELP in the study. This familiarity with the patients’ primary language and cultural norms likely eased communication and may have minimized care discrepancies for this patient population. A sensitivity analysis, which did not include Mandarin- and Cantonese-speaking patients from the NELP group, was therefore performed to assess the impact of NELP in the subgroup of patients who did not have this language and culturally specific support system. Similar to the overall analysis, time to surgery remained significantly longer for this subset of patients with NELP compared with patients with ELP, but survival outcomes were not significantly different. Notably, a breast cancer–specific nurse navigator was available for all patients, which could have helped preserve outcomes more broadly; however, this person was not fluent in languages other than English. Prior studies^8,13,18^ have demonstrated that institutional policies aimed at improving communication, including the use of professional interpreters and bilingual staff, can help reduce disparities in health outcomes for NELP populations. These findings highlight the critical role of system-level interventions in ensuring equitable care delivery, particularly in diverse health care settings. Strengthening and expanding culturally and linguistically tailored support services may further enhance access to timely care and improve outcomes for patients with NELP.

Despite the similar survival outcomes, the observed differences in socioeconomic factors and access to care highlight persistent inequities that could influence long-term health outcomes beyond the scope of our study. Patients with NELP in our cohort were more likely to have Medicaid insurance and reside in lower-income neighborhoods, factors that have been associated with reduced access to high-quality care and poorer cancer outcomes.^7,19,20^ Additionally, the significantly longer delays from diagnosis to surgery in the NELP group, particularly among patients from low-income communities, suggest that systemic barriers, such as difficulties in navigating the health care system or securing timely appointments, may still exist. These delays are concerning, as prior research has linked prolonged time to treatment initiation with worse prognosis for breast cancer.^17,21,22,23,24^

The results of this study emphasize that language preference alone may not drive disparities in breast cancer outcomes when robust support systems are in place. However, addressing the broader social and structural determinants of health that disproportionately affect patients with NELP remains critical. Interventions aimed at reducing treatment delays, improving health literacy, and ensuring culturally sensitive communication could further enhance outcomes for this vulnerable population.

Future research should explore the role of language barriers in other cancer-related end points, such as quality of life, patient satisfaction, and adherence to follow-up care, to provide a more comprehensive understanding of the impact of NELP on the cancer care experience. Additionally, investigating the effectiveness of targeted interventions, such as language-concordant care teams and digital health tools in multiple languages, could offer insights into scalable strategies for improving equity in cancer care.

### Limitations

Several limitations of this study should be noted. The retrospective design may introduce selection bias. Additionally, our findings are based on data from a single institution with a strong commitment to addressing language barriers. Thus, the generalizability to other health care settings, particularly those with fewer resources for patients with NELP, may be limited. The classification of NELP also relied on patient self-identification and a preference for interpreter services, which may not fully capture the variability in language proficiency or communication needs of all patients. Further, the data available did not contain information regarding receipt of neoadjuvant chemotherapy, which could affect time between diagnosis and surgery; however, disease stage, biomarker status, and grade as well as receipt of chemotherapy were similar between the 2 patient groups, suggesting that treatment patterns should be similar. Last, while clinical features known to be associated with cancer-related outcomes were used to adjust for confounding factors, additional confounders related to unmeasured social determinants of health may still exist.

## Conclusions

This cohort study demonstrates that NELP was not associated with worse breast cancer survival outcomes in a setting with dedicated resources for patients with NELP. However, the disparities in socioeconomic factors and treatment delays observed among patients with NELP highlight ongoing challenges in achieving equity in breast cancer care. Comprehensive, culturally sensitive strategies are needed to address these disparities and ensure all patients receive optimal cancer care, regardless of language preference.

## Notes

Supplement.
Data Sharing Statement
