# Supplementary material for: Non–English Language Preference and Breast Cancer Outcomes
Source: JAMA Netw Open. 2025 Jun 5;8(6):e2514036. doi: 10.1001/jamanetworkopen.2025.14036 (PMC12142445; doi:10.1001/jamanetworkopen.2025.14036)
Supplement: Supplement. — Data Sharing Statement [file jamanetwopen-e2514036-s001.pdf]

Data Sharing Statement

Spiegel. Non–English Language Preference and Breast Cancer Outcomes. *JAMA Netw Open*. Published June 05, 2025. doi:10.1001/jamanetworkopen.2025.14036

Data
Data available: No

Additional Information
Explanation for why data not available: Data will not be shared due to patient privacy.
